# Associations Between Pre‐Existing Cardiovascular Disease and Survival in Patients on Immune Checkpoint Inhibitor Therapy

**DOI:** 10.1002/cam4.70846

**Published:** 2025-04-28

**Authors:** Aditya Mahadevan, Aidan Vosooghi, Jagmeet Arora, Jeffrey Jones, Maziar Moslehyazdi, Warren A. Chow

**Affiliations:** ^1^ Department of Medicine University of California San Francisco San Francisco California USA; ^2^ Department of Internal Medicine University of Michigan Ann Arbor Michigan USA; ^3^ University of California Irvine School of Medicine Irvine California USA; ^4^ Division of Hematology/Oncology University of California Irvine Health Orange California USA

**Keywords:** cardiovascular disease, diastolic heart failure, immune checkpoint inhibitor, myocardial infarction, survival analysis, systolic heart failure

## Abstract

**Background:**

The impact of baseline cardiovascular disease (CVD) on survival in patients undergoing immune checkpoint inhibitor (ICI) therapy is not well understood. Therefore, we sought to determine the relationship between baseline CVD on mortality in patients undergoing ICI monotherapy or combination therapy.

**Methods:**

Using TriNetX, a global database of over 120 million patients, we identified 27,820 patients with pre‐existing cardiovascular disease prior to starting ICI monotherapy and an equal number of corresponding matched controls.

**Results:**

Systolic heart failure (HR: 1.38, 95% CI: 1.29–1.48), diastolic heart failure (HR: 1.34, 95% CI: 1.27–1.42), and atrial fibrillation/flutter (HR: 1.24, 95% CI: 1.19–1.29) had the greatest associations with mortality across ICI monotherapy.

**Conclusion:**

Future trials of patients initiating ICI therapy should capture these baseline values to guide risk assessment, pretreatment optimization, and surveillance strategies prior to treatment initiation.

## 1 Introduction

Since FDA approval of ipilimumab therapy in 2011 for the treatment of metastatic melanoma, the role of immune checkpoint inhibitor (ICI) therapy has grown rapidly [1, 2]. ICI‐related toxicity, or immune‐related adverse events (irAE), can manifest in virtually any organ and is believed to occur from hyperactivation of the immune system via PD‐1, PD‐L1 blockade, or CTLA‐4 blockade [3]. While cardiovascular irAEs and their risk factors have been studied extensively, equally important is the contribution of pre‐existing cardiovascular disease (CVD) to not only developing cardiovascular irAEs, but also ICI‐related morbidity and mortality. However, CVD and associated risk factors are underreported in ICI clinical trials, despite the fact that cancer and CVD share numerous bidirectional risk factors [4]. Clinical trials of ICIs frequently exclude patients with pre‐existing CVD, and in those trials that include such patients, the prevalence and composition of CVD are seldom reported. A systematic review of 69 ICI trials for cancer therapy identified zero trials reporting pre‐existing CVD; one study reported rates of hypertension, diabetes, or dyslipidemia, and one study reported pre‐existing cerebrovascular disease [5]. Despite compelling evidence that pre‐existing CVD may be linked to increased risk of cardiovascular irAEs and progression of cardiovascular disease, the association between pre‐existing CVD and mortality in patients receiving ICI therapy is not well understood. As such, this study aims to investigate the impact of pre‐existing CVD on survival in patients undergoing anti‐PD‐1/PDL‐1 and/or anti‐CTLA‐4 therapy across a global multi‐institutional cohort.

## 2 Methods

We utilized TriNetX, a global collaborative network comprising over 120 million patients, to identify cohorts of individuals with pre‐existing cardiovascular disease who received ICI therapy for any of the following malignancies commonly treated with ICI therapy: Malignant melanoma of the skin, malignant neoplasms of digestive organs, malignant neoplasms of the bronchus and lung, or malignant neoplasms of the urinary tract (Table S1). Monotherapy with ICI therapy was classified as patients receiving atezolizumab, avelumab, pembrolizumab, nivolumab, cemiplimab, or durvalumab. Combination therapy with ICI therapy was defined as patients receiving one of the previously mentioned monotherapy agents in combination with ipilimumab. All the following analyses were completed for patients receiving monotherapy and combination therapy. For the evaluation of pre‐existing cardiovascular disease, the experimental cohort consisted of patients diagnosed with any pre‐existing cardiovascular disease before the initiation of ICI therapy (Table S1). The control cohort consisted of patients without any pre‐existing cardiovascular disease receiving ICI therapy as defined by the same criteria. Additional analyses were conducted for patients with a single pre‐existing cardiovascular condition, such as angina pectoris, before starting ICI treatment and compared to the control cohort.

Prior to analyses, cohorts were 1:1 propensity score‐matched, controlling for age, sex, race, ethnicity, type of malignancy, the presence of distant metastases to account for cancer stage using the secondary cancer ICD‐10 code, and key risk factors for cardiovascular disease, including type 2 diabetes mellitus, hypertension, hyperlipidemia, smoking, chronic kidney disease, and overweight/obesity. The index event was defined as the first day of ICI treatment. The primary outcome of interest was mortality within 5 years of the index event. Kaplan–Meier survival analyses, including log‐rank tests and hazard ratios, were conducted using the TriNetX platform to compare mortality between the cohorts. The Benjamini‐Hochberg procedure was used to adjust for multiple comparisons when analyzing individual cohorts of pre‐existing cardiovascular disease.

## 3 Results

Our study identified 27,820 patients with pre‐existing cardiovascular disease who received anti‐PD‐1 or anti‐PD‐L1 therapy for malignant neoplasms of the bronchus and lung, digestive organs, urinary tract, or skin and an equal number of matched controls (*n* = 27,820). For patients receiving ICI combination therapy, 3320 patients with pre‐existing cardiovascular disease were identified with an equal number of matched controls (*n* = 3320) (Table 1).

**TABLE 1:** Demographics of patients treated with ICI therapy with and without pre‐existing cardiovascular disease.

Baseline characteristic	Monotherapy			Combination therapy		
	ICI with pre‐existing cardiovascular disease	ICI without pre‐existing cardiovascular disease	*p*	ICI with pre‐existing cardiovascular disease	ICI without pre‐existing cardiovascular disease	*p*
Total number of patients	27,820	27,820		3320	3320	
Age						
Mean (SD), years	68.8 (10.1)	68.5 (9.9)	0.0047	66.6 (10.9)	66.3 (10.3)	0.2213
Sex, *n* (%)						
Male	16,597 (59.7)	16,426 (59.0)	0.14	2236 (67.3)	2221 (66.9)	0.6952
Female	10,149 (36.5)	10,372 (37.3)	0.0501	941 (28.3)	954 (28.7)	0.7239
Race and ethnicity, *n* (%)						
White	18,805 (67.6)	18,855 (67.8)	0.6504	2455 (73.9)	2487 (74.9)	0.368
Not Hispanic or Latino	19,598 (70.4)	19,923 (71.6)	0.0024	2467 (74.3)	2504 (75.4)	0.2952
Black or African American	2506 (9.0)	2524 (9.1)	0.7902	176 (5.3)	164 (4.9)	0.5041
Asian	1531 (5.5)	1612 (5.8)	0.1369	141 (4.2)	126 (3.8)	0.3487
Cancer type, *n* (%)						
Malignant neoplasm of bronchus and lung	14,266 (51.3)	14,313 (51.4)	0.6901	913 (27.5)	883 (26.6)	0.4072
Malignant neoplasms of digestive organs	6961 (25.0)	7088 (25.5)	0.2152	593 (17.9)	597 (18.0)	0.8982
Malignant neoplasms of urinary tract	5546 (19.9)	5582 (20.1)	0.7028	989 (29.8)	992 (29.9)	0.9359
Malignant melanoma of skin	2642 (9.5)	2647 (9.5)	0.9424	1328 (40)	1352 (40.7)	0.5483
Secondary malignant neoplasm of other and unspecified sites	10,029 (36.1)	10,334 (37.1)	0.0073	1801 (54.2)	1841 (55.4)	0.3239
Comorbidity, *n* (%)						
Chronic kidney disease	4221 (15.2)	4156 (14.9)	0.441	569 (17.1)	562 (16.9)	0.8192
Hyperlipidemia	11,801 (42.4)	11,677 (42.0)	0.2871	1508 (45.4)	1516 (45.7)	0.8437
Hypertension	18,082 (65.0)	18,311 (65.8)	0.0413	2279 (68.6)	2325 (70.0)	0.2208
Overweight and obesity	5148 (18.5)	5113 (18.4)	0.702	787 (23.7)	804 (24.2)	0.625
Nicotine dependence	7804 (28.1)	7852 (28.2)	0.6509	648 (19.5)	641 (19.3)	0.8281
Type 2 diabetes mellitus	6640 (23.9)	6647 (23.9)	0.9445	872 (26.3)	834 (25.1)	0.2858

**FIGURE 1:**
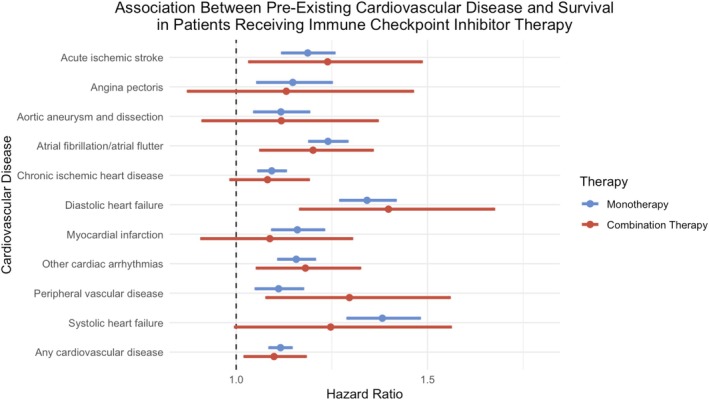
Association between pre‐existing cardiovascular disease and survival in patients on immune checkpoint inhibitor therapy.

The median survival for patients with pre‐existing cardiovascular disease on ICI monotherapy was 744 days with a survival probability of 33.5%. In comparison, the median survival for patients without pre‐existing cardiovascular disease was 913 days, with a survival probability of 38.2%. The presence of any pre‐existing cardiovascular disease in patients receiving anti–PD–1 or anti–PD‐L1 therapy was significantly associated with mortality (HR: 1.12, 95% CI: 1.09–1.14, *p* < 0.001) (Figure 1).

The median survival for patients with pre‐existing cardiovascular disease on ICI combination therapy was 789 days with a survival probability of 36.1%. In comparison, the median survival for patients without pre‐existing cardiovascular disease was 942 days, with a survival probability of 38.9%. The presence of any pre‐existing cardiovascular disease in patients receiving combination therapy was significantly associated with mortality (HR: 1.10, 95% CI: 1.02–1.18 *p* = 0.0098) (Figure 1).

For patients on ICI monotherapy, patients with systolic heart failure (HR: 1.38; 95% CI: 1.29–1.48, *p* < 0.001), diastolic heart failure (HR: 1.34, 95% CI: 1.27–1.42, *p* < 0.001), and atrial fibrillation/atrial flutter (HR: 1.24, 95% CI: 1.19–1.29, *p* < 0.001) had the highest risks of mortality. Lower risks of mortality, although statistically significant, were observed in patients with acute ischemic stroke (HR: 1.19, 95% CI: 1.12–1.26, *p* < 0.001), angina pectoris (HR: 1.15, 95% CI: 1.06–1.25, *p* = 0.0012), aortic aneurysm and dissection (HR: 1.12, 95% CI: 1.05–1.19, *p* = 0.0007), chronic ischemic heart disease (HR: 1.09, 95% CI: 1.06–1.13, *p* < 0.001), myocardial infarction (HR: 1.16, 95% CI: 1.10–1.23, *p* < 0.001), other cardiac arrhythmias (HR: 1.16, 95% CI: 1.11–1.21, *p* < 0.0001), and peripheral vascular disease (HR: 1.11, 95% CI: 1.05–1.17, *p* = 0.0002) (Table S2).

For patients on ICI combination therapy, patients with peripheral vascular disease (HR: 1.30, 95% CI: 1.08–1.56, *p* = 0.0053), diastolic heart failure (HR: 1.40, 95% CI: 1.17–1.67, *p* = 0.0002), and acute ischemic stroke (HR: 1.24, 95% CI: 1.04–1.48, *p* = 0.0195) had the highest risks of mortality. Lower risks of mortality, although statistically significant, were seen in patients with atrial fibrillation/atrial flutter (HR: 1.20, 95% CI: 1.08–1.78, *p* = 0.015) and other cardiac arrhythmias (HR: 1.18, 95% CI: 1.06–1.32, *p* = 0.0039) (Table S2).

## 4 Discussion

Our study is the first to investigate the association between pre‐existing cardiovascular disease and survival in patients receiving ICI monotherapy and combination therapy across a global, multi‐institutional cohort of nearly 30,000 patients. We found that across all categories of pre‐existing cardiovascular disease, patients with pre‐existing CVD had significantly increased rates of mortality, even when controlling for demographic characteristics, tumor type, and cancer stage.

Patients who experienced myocardial infarction (MI) at any point before initiation of ICI monotherapy demonstrated significantly higher rates of mortality. Prior retrospective studies have demonstrated an increased risk of MI and other major atherosclerotic cardiac events in patients undergoing ICI therapy [6, 7]. Smaller, single‐center studies to date have investigated the association between pre‐existing MI and clinical outcomes in patients undergoing ICI therapy, finding higher rates of adverse cardiovascular events in these patient populations [6, 8]. ICI‐related morbidity in these patients may be linked to accelerated atherosclerosis, which has already been demonstrated in patients on ICI therapy, potentially owing to several different mechanisms. ICIs may lead to the inhibition of negative regulatory pathways of atherosclerosis with a subsequent increase in plaque volume [9]. In addition, compared to patients with cancer not treated with ICI therapy, the inflammatory composition of coronary plaque in patients treated with ICIs has been shown to favor a lymphocyte‐predominant phenotype with an increased CD3/CD68 ratio, which may lead to plaque progression and instability [10]. This lymphocytic infiltration has also been suggested to cause increased arterial inflammation in patients on ICI therapy, particularly in patients without inflammatory lesions at baseline and in those without calcified lesions [11]. Clinically, atherosclerosis resulting from ICI therapy may play a key role in the development of subsequent ischemic events, as seen in the Javelin 101 trial in which 2% of patients on ICI experienced acute MI within 1 year of treatment [12, 13]. Consistent with this assertion, patients with pre‐existing autoimmune diseases, strongly linked to atherosclerotic heart disease, have demonstrated significantly higher rates of ischemic cardiovascular irAEs. Taken with prior literature, our findings suggest that patients with pre‐existing atherosclerotic cardiovascular disease and subsequent MI may be particularly vulnerable to further ischemic atherosclerotic events, as evidenced by significantly higher rates of mortality on ICI therapy compared to their counterparts without CVD.

Both systolic and diastolic heart failure were identified as significant risk factors for mortality following the initiation of ICI therapy. The interplay between cancer, cancer therapies, and heart failure is complex and multidirectional. Prior studies have demonstrated that patients with advanced cancer may develop cardiac wasting with accompanying decreases in left ventricular mass and function, a phenomenon referred to as cardiac wasting‐associated cardiomyopathy [14, 15, 16, 17, 18]. In the setting of advanced cancer and cancer‐related whole‐body cachexia, patients with existing heart failure may be more susceptible to worsening cardiac performance, functional status, and ultimately mortality. Further investigation is needed to understand the dynamic interplay between cancer and heart failure to elucidate causative mechanisms and therapeutic targets.

Hypertension is a key risk factor for diastolic heart failure, and while controlled for in our analyses, it may be a risk factor for poorer outcomes in patients on ICI therapy. Hypertension has been reported in a recent meta‐analysis as a common cardiovascular risk factor among patients who developed cardiovascular irAEs, including myocarditis [19]. Baseline hypertension has also been strongly associated with a composite outcome of myocardial infarction, coronary revascularization, and ischemic stroke. These findings contribute to the overall body of literature highlighting hypertension as an important risk factor to assess in patients who may be candidates for ICI therapy. ICI therapy has not been consistently shown to cause new‐onset hypertension, as demonstrated in a recent meta‐analysis, though the risk may be higher in patients who receive combination therapy [20]. Nevertheless, routine blood pressure monitoring represents a noninvasive and pragmatic monitoring target for patients undergoing treatment with ICIs. Given that hypertension may be a modifiable risk factor for the development of severe cardiovascular irAEs, our findings highlight the importance of serial blood pressure checks in patients receiving ICI therapy [21].

Patients with pre‐existing atrial fibrillation, flutter, and other cardiac arrhythmias were found to have worse outcomes in both the ICI monotherapy and combination therapy cohorts. Prior literature has demonstrated that ICI therapy can cause arrhythmias, often in combination with other cardiotoxicities, with more severe arrhythmias occurring within 30 days of ICI administration [22, 23]. However, outcome data in patients with pre‐existing arrhythmias receiving ICI therapy is limited. While cause‐specific mortality in our study population is difficult to ascertain, our findings suggest an important role for pre‐treatment optimization of these arrhythmias. This is particularly important given that patients on ICI therapy may develop irAEs that could exacerbate pre‐existing arrhythmias, including ICI‐related thyroid dysfunction, myocarditis, or pericarditis [24, 25, 26].

Our all‐sum cohort of patients experiencing pre‐existing CVD demonstrated higher rates of mortality than patients without pre‐existing CVD, despite adjusting for several key clinical and demographic factors. Of note, we did not evaluate cause‐specific mortality as part of these analyses. However, there are several plausible mechanisms for increased mortality in this population. For one, patients with pre‐existing cardiovascular disease may be more likely to develop major adverse cardiovascular events (MACE), as demonstrated in a retrospective single‐center analysis [27]. ICI‐related MACE is likely driven by several factors, including the aforementioned T‐cell mediated vascular infiltration and atherosclerosis, as well as PD‐1 and CTLA‐4 mediated myocardial injury [28, 29, 30, 31]. Further research is needed to elucidate cause‐specific mortality in this patient population to risk stratify these patients and ultimately mitigate adverse events.

This study's limitations include its retrospective nature, usage of claims data reliant on ICD‐10 coding, and the absence of cause‐specific mortality. ICD‐10 coding inherently cannot fully capture the complexity and nuance of cardiovascular disease, nor is it able to fully capture inherent differences between patients, such as lifestyle factors or baseline medication use. However, the ICD‐10 codes in our study were chosen based on careful literature review and by other previously published TriNetX studies. Additionally, as this data was sourced from multiple global databases through TriNetX, inherent differences in medical practices, treatment plans, as well as genetic differences by region may affect the generalizability of the research results. Nevertheless, the high rates of observed statistical significance suggest that these trends hold true across a variety of settings.

## 5 Conclusions

The increased risk of mortality from pre‐existing CVD we observed highlights the need for further inclusion of these baseline characteristics in future clinical trials involving immune checkpoint inhibitors. Our work also highlights the paramount importance of developing more robust screening and mitigation measures for patients at risk of developing cardiovascular events during and after ICI treatment. Such events may lead to mortality directly or may threaten cancer‐related survival due to interruptions in life‐saving treatment. Accordingly, future studies should further investigate cause‐specific mortality in patients with cardiovascular disease and comorbidities receiving ICI therapy, as well as mortality resulting directly from cardiovascular irAEs.

## Author Contributions

All authors contributed to the paper and consented to be added as co‐authors. A.M.: Conceptualization, investigation, writing – original draft, methodology, writing – review and editing. A.V.: Conceptualization, investigation, writing – original draft, methodology, writing – review and editing. J.A.: Conceptualization, investigation, writing – original draft, methodology, writing – review and editing, formal analysis, data curation. J.J.: Methodology, writing – review and editing, formal analysis, data curation. M.M.: Methodology, writing – review and editing, formal analysis, data curation. W.A.C.: Conceptualization, investigation, writing – review and editing, project administration, supervision.

## Ethics Statement

Ethical approval was not required for this study, as it did not involve personally identifiable data. The research was conducted in accordance with all applicable regulations and no IRB was required.

## Consent

The authors have nothing to report.

## Conflicts of Interest

The authors declare no conflicts of interest.

## Supporting information


Figure S1.

Figure S2.

Table S1.

Table S2.

## Data Availability

The data that support the findings of this study are available from TriNetX. Restrictions apply to the availability of these data, which were used under license for this study. Data are available from the author(s) with the permission of TriNetX.
