# Supplementary material for: Associations Between Pre‐Existing Cardiovascular Disease and Survival in Patients on Immune Checkpoint Inhibitor Therapy
Source: Cancer Med. 2025 Apr 28;14(9):e70846. doi: 10.1002/cam4.70846 (PMC12037690; doi:10.1002/cam4.70846)

Supplemental Figure 2: Kaplan Meier curve of patients receiving ICI combination therapy with versus without pre-existing cardiovascular disease. The purple curve denotes the survival probability of patients with pre-existing cardiovascular disease. The green curve denotes the survival probability of patients without pre-existing CVD.


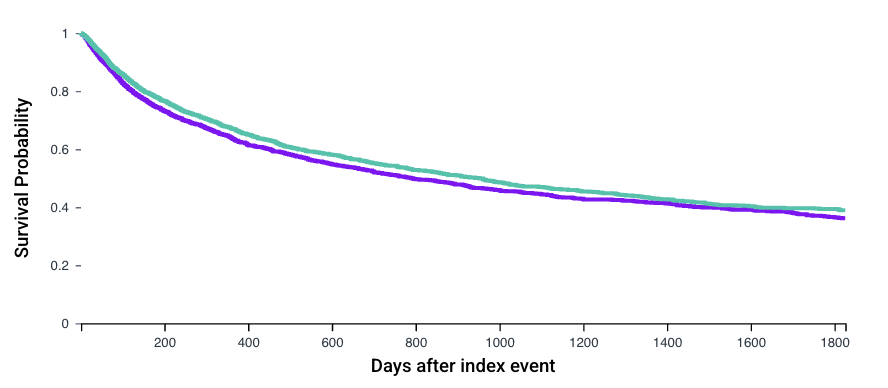

Supplement: Supplementary file 2 — Figure S2. [file CAM4-14-e70846-s001.docx]
