# Supplementary material for: Associations Between Pre‐Existing Cardiovascular Disease and Survival in Patients on Immune Checkpoint Inhibitor Therapy
Source: Cancer Med. 2025 Apr 28;14(9):e70846. doi: 10.1002/cam4.70846 (PMC12037690; doi:10.1002/cam4.70846)
Supplement: Supplementary file 3 — Table S1. [file CAM4-14-e70846-s003.docx]

| **Cardiovascular Disease** | **ICD-10 Code** |
| --- | --- |
| Atrial fibrillation/Atrial flutter | I48 |
| Angina pectoris | I20.9 |
| Aortic aneurysm and dissection | I71 |
| Acute ischemic stroke | I63 |
| Chronic ischemic heart disease | I25 |
| Diastolic heart failure | I50.3/I11.0 |
| Myocardial infarction | I21/I22 |
| Other cardiac arrhythmias | I49 |
| Peripheral vascular disease | I73.9 |
| Systolic heart failure | I50.2 |
| **Cardiovascular Risk Factor** | **ICD-10 Code** |
| Chronic kidney disease | N18 |
| Essential (primary) hypertension | I10 |
| Hyperlipidemia, unspecified | E78.5 |
| Nicotine dependence | F17 |
| Overweight and obesity | E66 |
| Type 2 diabetes mellitus | E11 |
| **Malignancy** | **ICD-10 Code** |
| Malignant neoplasms of digestive organs (C15-26) |  |
| Malignant neoplasm of esophagus | C15 |
| Malignant neoplasm of stomach | C16 |
| Malignant neoplasm of small intestine | C17 |
| Malignant neoplasm of colon | C18 |
| Malignant neoplasm of rectosigmoid junction | C19 |
| Malignant neoplasm of rectum | C20 |
| Malignant neoplasm of anus and anal canal | C21 |
| Malignant neoplasm of liver and intrahepatic bile ducts | C22 |
| Malignant neoplasm of gallbladder | C23 |
| Malignant neoplasm of other and unspecified parts of biliary tract | C24 |
| Malignant neoplasm of pancreas | C25 |
| Malignant neoplasm of other and ill-defined digestive organs | C26 |
| Malignant neoplasm of bronchus and lung | C34 |
| Malignant melanoma of the skin | C43 |
| Malignant neoplasms of the urinary tract (C64-68) |  |
| Malignant neoplasm of kidney, except renal pelvis | C64 |
| Malignant neoplasm of renal pelvis | C65 |
| Malignant neoplasm of bladder | C66 |
| Malignant neoplasm of other and unspecified urinary organs | C68 |

Supplemental Table 1: ICD-10 codes
