# Supplementary material for: Associations Between Pre‐Existing Cardiovascular Disease and Survival in Patients on Immune Checkpoint Inhibitor Therapy
Source: Cancer Med. 2025 Apr 28;14(9):e70846. doi: 10.1002/cam4.70846 (PMC12037690; doi:10.1002/cam4.70846)
Supplement: Supplementary file 4 — Table S2. [file CAM4-14-e70846-s002.docx]

|  | **Monotherapy** | | | **Combination Therapy** | | |
| --- | --- | --- | --- | --- | --- | --- |
| **Condition** | **Hazard ratio (95% CI)** | **p-value** | **Sample size** | **Hazard ratio (95% CI)** | **p-value** | **Sample size** |
| Acute ischemic stroke | 1.19 (1.12-1.26) | <0.001 | 5,133 | 1.24 (1.04-1.48) | 0.0195 | 498 |
| Angina pectoris | 1.15 (1.06-1.25) | 0.0012 | 2,422 | 1.13 (0.88-1.46) | 0.3469 | 250 |
| Aortic aneurysm and dissection | 1.12 (1.05-1.19) | 0.0007 | 4,102 | 1.12 (0.91-1.37) | 0.2812 | 384 |
| Atrial fibrillation/Atrial flutter | 1.24 (1.19-1.29) | <0.001 | 10,827 | 1.20 (1.06-1.36) | 0.003 | 1,118 |
| Chronic ischemic heart disease | 1.09 (1.06-1.13) | <0.001 | 17,116 | 1.08 (0.99-1.19) | 0.097 | 1,935 |
| Diastolic heart failure | 1.34 (1.27-1.42) | <0.001 | 5,759 | 1.40 (1.17-1.67) | 0.0002 | 501 |
| Myocardial infarction | 1.16 (1.10-1.23) | <0.001 | 5,150 | 1.09 (0.91-1.30) | 0.3546 | 528 |
| Other cardiac arrhythmias | 1.16 (1.11-1.21) | <0.001 | 10,504 | 1.18 (1.06-1.32) | 0.0039 | 1,302 |
| Peripheral vascular disease | 1.11 (1.05-1.17) | 0.0002 | 5,443 | 1.30 (1.08-1.56) | 0.0053 | 436 |
| Systolic heart failure | 1.38 (1.29-1.48) | <0.001 | 3,523 | 1.25 (1.00-1.56) | 0.052 | 313 |
| Any pre-existing cardiovascular disease | 1.12 (1.09-1.14) | <0.001 | 27,820 | 1.10 (1.02-1.18) | 0.0098 | 3,320 |

Supplemental Table 2: Hazard Ratios (95% CI) and p-values for cardiovascular diseases in patients treated with ICI monotherapy or combination therapy
